# The Characteristics and Distribution of α2D-, α2B- and α2C-Adrenoceptor Subtypes in Goats

**DOI:** 10.3390/ani12050664

**Published:** 2022-03-07

**Authors:** Ming Xu, Qiulin Zhang, Qi Wang, Di Pan, Mingxing Ding, Yi Ding

**Affiliations:** College of Veterinary Medicine, Huazhong Agricultural University, Wuhan 430070, China; mingxu1101993@sina.com (M.X.); colynqiulin@163.com (Q.Z.); meixianling@yeah.net (Q.W.); zsyxhtg@163.com (D.P.); dmx@mail.hzau.edu.cn (M.D.)

**Keywords:** α2 adrenoceptor subtypes, intracerebroventricular injection, intrathecal injection, analgesia, goat

## Abstract

**Simple Summary:**

α2-Adrenergic receptors mediate many diverse biological effects of the endogenous catecholamines epinephrine and norepinephrine. Three distinct subtypes of α2-adrenergic receptors, α2B, α2C and α2D, have been identified in goats; however, the characteristics and distribution of α2-adrenoceptors in goats remain unclear. Therefore, the aim of our study was to assess the characteristics and distribution of α2-adrenoceptor subtypes in goats. Our study highlights the wide but uneven distribution of α2-adrenoceptor subtypes in goats. Additionally, our study showed that α2D-ceptor has a better analgesic effect in goats than α2B- and α2C-adrenoceptor, whereas α2C-adrenoceptor plays a more important role in thermoregulation than α2B- and α2D-adrenoceptors.

**Abstract:**

α2-Adrenegic receptors (α2Rs) are important presynaptic modulators of central noradrenergic function (auto receptors) and postsynaptic mediators of many of the widespread effects of catecholamines and related drugs. Studies have shown that ruminants (such as goats and cattle) express special α2DR subtypes in addition to α2BR and α2CR. Real-time quantitative PCR and Western blotting were used to investigate the distribution and density of α2R in different nuclei of the goat central nervous system, selected regions of the spinal cord (L4-L6), and in various peripheral tissues. α2-AR subtype-specific antibodies were injected intrathecally and intracerebroventricularly into the tested goats to block the corresponding subtype of receptors. Pain threshold and physiological parameters were evaluated to explore the functional characteristics of α2BR, α2CR and α2DR in goats. Our results suggest that the expression of the mRNAs and proteins of all three α2R subtypes are widely but unevenly distributed in the goat CNS and peripheral tissues. Furthermore, α2DR plays a more important role in α2R-mediated analgesia in goats than α2BR and α2CR, whereas α2CR activation exerts a greater effect on body temperature than α2BR and α2DR.

## 1. Introduction

α2-Adrenergic receptor (α2R) agonists are some of the most commonly used sedative analgesic drugs in veterinary medicine due to their availability, reversibility and rapid onset [1,2,3]. This class of drugs is commonly used as a premedication to sedate animals or as an anesthetic adjuvant to reduce the side effects of other drugs. In ruminants, α2R agonists are often used alone in minor procedures, such as rectal exams, biopsies, or radiographs [4]. The combination of an α2R agonist with a local anesthetic is typically used in more painful procedures, such as flank surgery, castration, or wound management [5]. The side effects of α2R agonists include hypothermia, decreased heart rate and biphasic effects on blood pressure, which manifest as transiently increased blood pressure at an early stage and decreased blood pressure at a later stage [6].

Mechanistically, α2R agonists exert their effects by reacting with presynaptic α2R in the central nervous system and reducing neurotransmitter release through a negative feedback mechanism [7]. Previous studies have implicated the locus coeruleus, a major nucleus in the central nervous system, in the control of the vigilance and initiation of behavioral responses [8]. The sedative effect of α2R agonists results from the suppression of neuronal signals from the locus coeruleus [9]. The analgesic effect of α2R agonists was reported to be achieved through interference with ascending nociceptive pathways in the dorsal horn of the spinal cord [10]. The α2R agonist significantly decreases glutamate and substance p release from the dorsal horn of the spinal cord and thereby reduces nociceptive transmission. Supraspinal sites are also involved in α2R agonist-mediated analgesic effects; Peng et al. indicated that α2R in the periventricular gray is important for suppressing pain using the cell clamp technique [11]. Herbert et al. detected abundant α2R in the parabrachial nucleus [12]. As the parabrachial nucleus is a relay center for pain signal transmission, it was postulated to be an important target that mediates α2R agonist-induced analgesic effects. In addition, α2R agonists also activate postsynaptic α2R in effector organs and are mainly responsible for its cardiovascular effects [13]. Kästner et al. also reported that an α2R agonist activates pulmonary intravascular macrophages (PIMs), a specialized group of macrophages found in ruminants, which are capable of producing a range of vasoactive mediators and cytokines [14] One study showed the rapid onset of arterial hypoxemia and its complete reversal by al-pha2 antagonists given before or within the first 5 min after xylazine administration [15], indicating that functional changes such as vasoconstriction and the redistribution of blood flow are responsible for the first stage of impaired oxygenation. It was suggested that alterations in pulmonary hemodynamics with significantly increased mean pulmonary artery pressure, pulmonary arterial occlusion pressure and estimated capillary pressure constitute the initial stage of α2R agonist-induced pulmonary edema. The increased pressure may facilitate PIM activation and then lead to pulmonary edema and hypoxia [16].

α2Rs are a group of G protein-coupled receptors located on the cell membranes of both neural and nonneural tissues. Researchers have confirmed that α2Rs are heterogeneous receptors that consist of α2A, α2B, α2C and α2D subtypes, according to their pharmacological characteristics [14]. Ruminants express a special α2DR subtype, which is the orthologue of α2A subtypes in other species [15]. Only 45–55% similarity in the amino acid sequences of the α2A/D, α2B and α2C receptor subtypes has been observed. In addition, species differences in the expression and distribution of α2R subtypes have been identified; for example, the human spinal cord expresses both α2BR and α2CR [17], while the rat spinal cord mainly expresses α2CR [18]. Interestingly, the dosage of α2R agonist in ruminants is only one-tenth of that in other animals; however, a low dose of α2R agonist still induces significant side effects in ruminants, including a 50% decrease in cardiac output, hypothermia and hypotension [19]. Researchers have not yet determined whether this superior sedative and analgesic effect of α2R agonists on ruminants is attributed to the distribution and density of the α2R subtypes.

To the best of our knowledge, no scientific reports have documented the distribution and functional characteristics of α2R subtypes in ruminants. A previous study used a radiolabeled α2R agonist to investigate the distribution of α2R in ruminants; however, these α2R agonists are not truly subtype selective and do not differentiate subtypes. In the present study, we described the distribution, density and functional characters of α2R subtypes in goats, which would help develop α2R subtype-specific agonists that are more effective in analgesia and sedation, and exert fewer side effects on ruminants.

## 2. Materials and Methods

### 2.1. Animal Preparation

One- to two-year-old healthy local male goats (Yichang White Goat) weighing 23–28 kg were purchased from the Laboratory Animal Research Institute in Wuhan, China. All goats had free access to food and water and were dewormed and adapted to the environment for 7 days. The study was conducted under the ethical guidelines approved by the Institutional Animal Care and Use Committee of Huazhong Agricultural University (HZAUGO-2019-03) and adhered to the guidelines of the Committee for Research and Ethical Issues of the International Association for the Study of Pain.

### 2.2. Experimental Design

Thirty goats were used in this study. The goats were randomly divided into 5 groups (*n* = 6): nonspecific immunoglobulin (IgG) (Frdbio, Wuhan, China), yohimbine (Yoh) (Absin, Shanghai, China), α2BR antibody (α2BR Ab), α2CR antibody (α2CR Ab) and α2DR antibody (α2DR Ab) groups. Catheters were placed in the third ventricle and intrathecal space for 14 days. After 7 days of intubation, the same doses of α2DR-, α2BR- and α2CR-specific neutralizing antibody (30 μg/kg) (Frdbio, Wuhan, China); nonspecific control immunoglobulin (30 μg/kg); and the α2R nonselective antagonist yohimbine (3 μg/kg) were injected intrathecally or intracerebroventricularly in each group. After 30 min of injection, a low dose of xylazine (Absin, Shanghai, China) (0.05) mg/kg was injected intramuscularly, and the nociception threshold at different time points after the xylazine injection was statistically analyzed.

### 2.3. Catheter Implantation Procedure

The goats were anesthetized with isoflurane and placed in a stereotaxic frame. A stainless-steel cannula (RWD, Shenzhen, China) was sterotaxically introduced into the third ventricle of each goat, as described by Qiu et al. [20]. The position of the cannula tip was confirmed in the third ventricle when CSF flowed out. The cannula was then fixed on the skull with acrylic dental cement. A plastic cap was then placed on the top of the cannula to prevent backflow of the CNS fluid when injecting α2DR-, α2BR- and α2CR-specific neutralizing antibodies. The cap was removed, and a catheter was introduced into the cannulas for injection. For intrathecal catheter implant placement, stainless-steel cannulas were slowly inserted perpendicularly into the spinal column. Then, a catheter (RWD, Shenzhen, China) was slowly inserted approximately 10 cm into the intrathecal space through the lumen of the cannula. The cannula was then removed. The implanted catheter remained in place with 10 cm left outside the animal and stitched onto the animal. After catheter implant placement, the goats were administered 7000 U/kg penicillin via intramuscular injection daily for 4 consecutive days and then rested for two weeks.

### 2.4. Antibody Preparation

The gene sequences or corresponding deduced amino acid sequences of α2DR (XM_018041717.1, XP_017897206.1α), α2BR (XM_018054838.1, XP_017910327.1α) and α2CR (XM_013964836.2, XP_013820290.2α) were used as queries to search the National Center for Biotechnology Information database (https://www.ncbi.nlm.nih.gov/, accessed on 18 December 2018). The α2R subtypes from goats were aligned, and each subtype from human, mouse, cattle and goat was compared, as demonstrated in Figures S1–S4. The extracellular domains of the α2DR, α2BR and α2CR proteins were predicted with TMHMM Server V. 2.0 (http://www.cbs.dtu.dk/services/TMHMM/, accessed on 27 February 2022). The immunogenicity of the extracellular domain of the proteins was analyzed using DNAStar software (DNAStar Inc, Madison, WI, USA). The extracellular amino acid sequence that exhibited high immunogenicity was then determined to generate neutralizing antibodies. The sequences of anti-α2DR, anti-α2BR and anti-α2CR peptides were as follows: FRQEQPLAEGSFAPMGSLQPDAGC, DHQEPYSVQATAAC and GAPWGPPPGQYSAGC, respectively. The peptides were synthesized and purified by Frdbio (Wuhan, China). Synthetic antigenic peptides were conjugated to keyhole limpet hemocyanin (KLH) protein, and each was used to immunize rabbits. Male New Zealand White rabbits (3 to 4 kg) were immunized subcutaneously with 500 μg of the conjugated protein in Freund’s complete adjuvant (Sigma-Aldrich Inc, St. Louis, MO, USA). Three booster injections were administered subcutaneously at 2-week intervals with the same amount of conjugated protein emulsified in Freund’s incomplete adjuvant. The rabbits were bled after the fourth immunization. The polyclonal antibodies against α2DR, α2BR and α2CR were then purified using Protein A affinity chromatography (Frdbio, Wuhan, China) for use in subsequent experiments.

### 2.5. Nociception Threshold Determination

The basic nociception threshold was measured before the intracerebroventricular or intrathecal injection. Goats were lightly tied to a padded rack. The goats were standing during drug injections and pain threshold testing. Changes in the nociception threshold were measured every 10 min after injections by recording potassium iontophoresis and the hindlimb withdrawal threshold (HWT) [21,22]. Before the measurement, the skin in the central area of the left abdomen of the goat was shaved, washed with soap and 75% alcohol, degreased and disinfected. When measuring the nociception threshold with the DL-ZII DC induction electrotherapy machine (Shantou Medical Equipment Factory Co., Ltd., Shantou, China), two acupuncture probe electrodes dipped in a saturated potassium chloride solution were adhered tightly to the left abdomen skin with an interval of approximately 2–3 cm. The voltage intensity was gradually increased and stopped when the tested skin and muscles started to tremble and shrink. The voltage intensity at this time point was measured as the goat nociception threshold.

The HWT method was used to measure the reflex nociception threshold. Before the measurement, the tension sensor (connected to the tactile measurement kit) (ZS Dichuang Science and Technology Development Co., Ltd., Beijing, China) was calibrated with a 30 g standard weight. A tactile measurement kit was used to stimulate the right hind leg of the goat slowly with the intensity increasing from light to strong. When the goat showed a retraction reflex, mechanical stimulation stopped automatically. At this time point, the intensity value measured by the tension sensor was determined as the nociception threshold.

After the xylazine injection, the nociception threshold was measured every 10 min and recorded as the nociception thresholds T_n_ and V_n_ at the time point (each method was repeated 3 times, with an interval of 2 min). Before the antibody injection, the mechanical nociception threshold and potassium iontophoresis nociception threshold were repeatedly measured three times as the basic values T_0_ and V_0_. A statistical analysis was performed to evaluate the nociception threshold at different time points after the xylazine injection.

### 2.6. Measurement of Physiological Indices

The basic physiological indicators of goat were measured before the intraventricular (intrathecal) drug injection and recorded every 10 min after the antibody was injected. Xylazine was injected 40 min after the antibody. We recorded the parameters for 1 h after the xylazine injection, i.e., the measurement in the whole process of the environment lasted 100 min. The physiological indicators, including body temperature, heart rate, respiratory rate, blood oxygen saturation and blood pressure, were measured 3 times, and the average values were calculated as the results for each time point. Blood oxygen saturation was monitored with a Mindray PM-9000 portable multiparameter ECG monitor (Mindray Biomedical Electronics Co., Ltd., Shenzhen, China). Heart rates were monitored with an electrocardiogram monitoring unit. Blood pressure was monitored with a blood pressure monitoring unit and a suitable size cuff, which connected the cuff vent and wrapped around the upper left knee joint of the goat. The sensor was placed in the position corresponding to the femoral artery of the goat. Blood pressure was measured three times at each time point, and the average of three blood pressure values was recorded as the measurement result. Blood oxygen saturation was monitored using a portable blood oxygen saturation meter. During the experiment, the photoelectric sensor (ear tongue clip) was clamped onto the ear of the tested goat, and the value was measured three times at each time point.

### 2.7. Sample Collection

**Figure 1:**
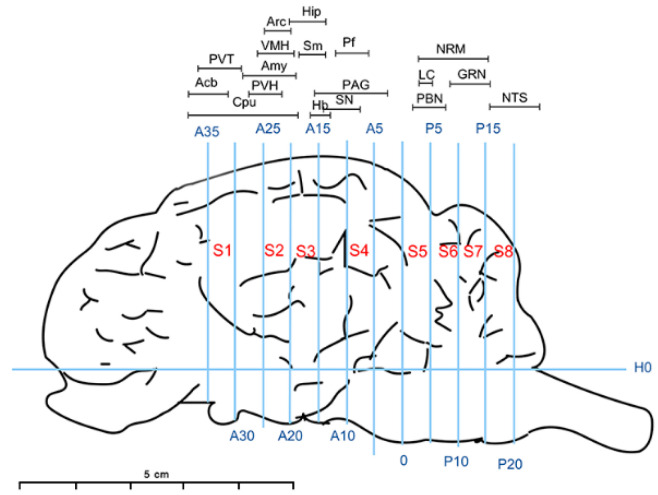
Brain section: H0: the horizontal zero plane. The 0 plane represents the interaural line. A35, A25, A20, A15, A10 and A5 show transverse planes located 35, 30, 25, 20, 15, 10 and 5 mm rostral to the interaural line, and P5, P10, P15 and P20 show transverse planes located 5, 10, 15 and 20 mm caudal to the interaural line, respectively. S1-8, take the base edge of the olfactory bulb as the starting line, and make a sagittal plane, each 5mm. The locations of nuclei or areas in the brain blocks are presented at the top of the figure. The nuclei and areas identified include the caudate putamen nucleus (Cpu), nucleus accumbens (Acb), paraventricular thalamus (PVT), hypothalamic paraventricular nucleus (PVN), arcuate nucleus (Arc), amygdala (Amy), ventromedial hypothalamic nucleus (VMH), hippocampus (Hip), thalamic nucleus submedius (Sm), habenular nucleus (Hb), parafascicular nucleus (Pf), substantia nigra (SN), periaqueductal gray (PAG), parabrachial nucleus (PBN), locus coeruleus (LC)), nucleus raphe magnus (NRM), gigantocellular reticular nucleus (GRN) and nucleus of the solitary tract (NTS).

Three goats were euthanized with 2,4-xylene thiazole. The goat brain and spinal cord L4-L6 were immediately removed from the skull and lumbar spinal canal, respectively. The nuclei and brain area were collected with 4–10 cm diameters in DEPC-treated plastic tubes based on protocols described in our previous work [20]. The following nuclei and regions related to pain signal transmission and modulation were selected in this study (Figure 1): forebrain: caudate putamen nucleus (Cpu), nucleus accumbens (Acb), paraventricular thalamus (PVT), hypothalamic paraventricular nucleus (PVN), arcuate nucleus (Arc), amygdala (Amy), ventromedial hypothalamic nucleus (VMH), hippocampus (Hip), thalamic nucleus submedius (Sm), habenular nucleus (Hb) and parafascicular nucleus (Pf); midbrain: substantia nigra (SN) and periaqueductal gray (PAG); hindbrain: parabrachial nucleus (PBN), locus coeruleus (LC), nucleus raphe magnus (NRM), gigantocellular reticular nucleus (GRN) and nucleus of the solitary tract (NTS); and spinal cord L4-L6 dorsal horn (SCDH).

Peripheral tissues, including the myocardium (Myo), liver, spleen, lung, kidney, aorta, superior vena cava (SVC), inferior vena cava (IVC), arterioles, venules and blood mononuclear macrophages (Mφs), were collected to investigate the distribution and density of α2DR, α2BR and α2CR.

Blood mononuclear macrophages were isolated from heparinized blood samples using the instructions provided in the goat peripheral blood mononuclear cell separation kit (P5290, Solarbio, Beijing, China). The cells obtained through density gradient centrifugation were resuspended in monocyte media at a density of 106 cells/mL, plated on cell culture plates and placed in a cell incubator for adherent culture. After 3 h, all nonadherent cells were removed, and the adherent cells were blood mononuclear macrophages.

### 2.8. RNA Isolation and Real-Time Quantitative PCR

After the tissue was sampled, it was ground in liquid nitrogen and treated with 1 mL of TRIzol (ThermoFisher Scientific, Waltham, MA, USA) reagent. Then, 200 μL of chloroform was added and incubated at 25 °C for 15 min. The sample mixture was centrifuged at 12,000× *g* for 10 min at 4 °C. The supernatant was mixed with an equal amount of isopropanol. After gentle inversion, the sample was frozen at −20 °C for 30 min and then centrifuged at 12,000× *g* for 10 min at 4 °C. Subsequently, the sample was washed twice with precooled 75% ethanol, and then DEPC water was added to dissolve the total RNA to 1000 ng/μL. Then, reverse transcription PCR was performed. Real-time qPCR was performed using SYBR^®^ Green I according to the manufacturer’s protocol (ABclonal Technology, RK21203, Woburn, MA, USA). The relative expression of the target gene relative to the housekeeping gene (GAPDH) was determined using the Cq method. The average value of the Cq values of different groups was used as ΔΔCt, and the relative quantification was calculated using the equation RQ = 2^−ΔΔCt^. All samples were measured in triplicate.

### 2.9. Protein Separation and Western Blot Analysis

The collected tissues were ground in liquid nitrogen, and the protein was extracted with RIPA buffer containing PMSF and phosphatase inhibitors for 30 min according to the manufacturer’s instructions (Beyotime, P0013B, Shanghai, China). The protein concentration was then analyzed using a BCA protein detection kit (Beyotime, P0010, Shanghai, China). Thirty micrograms of each protein sample was separated on a 10% SDS–PAGE gel and then transferred to a polyvinylidene fluoride membrane (Life Technologies, ThermoFisher Scientific, Waltham, MA, USA). After an incubation with 5% skim milk for 2 h at 25 °C, the membrane was immunolabeled with a primary antibody at 4 °C overnight. The membrane was washed with TBST buffer and then incubated with α2B-, α2C- and α2D-specific multiclonal antibodies for 2 h at 25 °C. After washes with TBST buffer, the membrane was treated with horseradish peroxidase substrate, and then the signal was detected with a chemiluminescence imaging system.

### 2.10. Statistical Analysis

All data were statistically analyzed using SPSS 18.0 software (SPSS Inc., Chicago, IL, USA). The data are presented as the means ± SD. The pain threshold and physiological indicator data were analyzed using two-way ANOVA followed by Bonferroni’s post hoc test. The decreases in body temperature were analyzed using one-way ANOVA followed by Bonferroni’s post hoc test. The least significant difference (LSD) test was used for multiple comparisons. * Indicates a significant difference between the indicated groups: * *p* < 0.05 and ** *p* < 0.013.

## 3. Results

### 3.1. Real-Time Quantitative PCR Analyses of α2DR, α2BR and α2CR mRNA Abundance in Selected CNS Nuclei and Regions, as well as Peripheral Tissues

**Figure 2:**
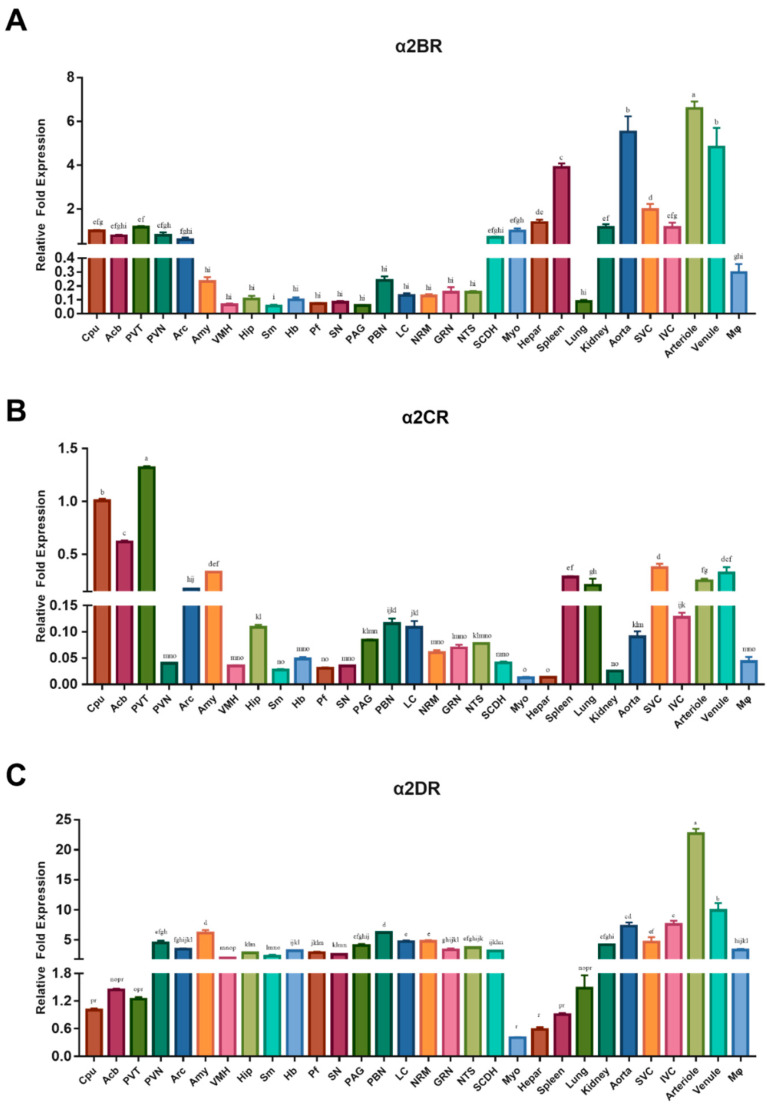
Real-time qPCR analyses of α2BR (**A**), α2CR (**B**) and α2DR (**C**) mRNA abundances in the Cpu, Acb, PVT, PVN, Arc, Amy, VMH, Hip, Sm, Hb, Pf, SN, PAG, PBN, LC, NRM, GRN, NTS, SCDH, Myo, liver, spleen, lung, kidney, aorta, SVC, IVC, arterioles, venules and Mφ of goats. The results were normalized to GAPDH as a housekeeping gene and are presented as the means ± SD (*n* = 3). Bars with different letters differ (*p* < 0.05).

The levels of the α2DR, α2BR and α2CR mRNAs were detected in different nuclei and regions in the CNS and peripheral tissues of goats to assess the distribution of α2DR, α2BR and α2CR in the CNS and peripheral tissues in goats (Figure 2). The relative fold change in the expression of each α2R mRNA within a specific CNS nucleus or peripheral tissue was calculated as the ratio of their expression to that of a housekeeping gene (GAPDH). The mRNAs encoding the receptor subtypes were widely but unevenly distributed in different tissues.

In pain-related nuclei, the α2BR mRNA (Figure 2A) was distributed at relatively high levels in the Cpu, Acb, PVT, PVN, Arc and SCDH. The α2BR mRNA was abundant in the following goat peripheral tissues: arteriole, spleen, aorta and venule (*p* < 0.05).

The mRNA encoding α2C (Figure 2B) in the Cpu and PVT was among the pain-related nuclei and regions. The Acb, Arc and Amy also exhibited high levels of α2C mRNA but lower levels than the Cpu and PVT. Among the peripheral tissues, a higher α2CR mRNA abundance was detected in the spleen, lung, SVC, arterioles and venule than Myo, hepatic, kidney, aorta and Mφ tissues (*p* < 0.5).

Figure 2C shows a more equal distribution of the α2DR mRNA between various nuclei in the CNS, with relatively lower levels in the Cpu, Acb and Pvt. The α2DR mRNA was most abundant in the goat arteriole among the peripheral tissues, and significant levels were also detected in the goat kidney, aorta, SVC, IVC, venule and Mφ (*p* < 0.5). 

Simultaneously, we also calculated and compared the relative levels of α2DR, α2BR and α2CR mRNAs within each selected CNS nucleus and peripheral tissue in goats (Figure S2). The α2DR mRNA was expressed at significantly higher levels in the PVN, Sm, Pf, PAG, LC, NRM, SCDH, kidney, aorta, IVC, arterioles and Mφ than in the α2BR and α2CR (*p* < 0.05). The α2CR mRNA was more abundant in the Cpu, Acb, PVT, Arc, Amy, Hip, spleen, lung, SVC and venule than α2BR and α2DR mRNAs (*p* < 0.05). Lower levels of the α2BR mRNA were detected in most central nervous tissues than levels of the α2CR and α2DR mRNAs, except for the GRN and SCDH. In peripheral tissues, the α2BR mRNA was expressed at higher levels than the α2DR mRNA in the spleen and lungs and at higher levels than both the α2CR and α2DR mRNAs in macrophages and the liver.

### 3.2. Western Blot Analyses of α2DR, α2BR and α2CR mRNA Abundances in Selected CNS Nuclei and Regions, as well as Peripheral Tissues

**Figure 3:**
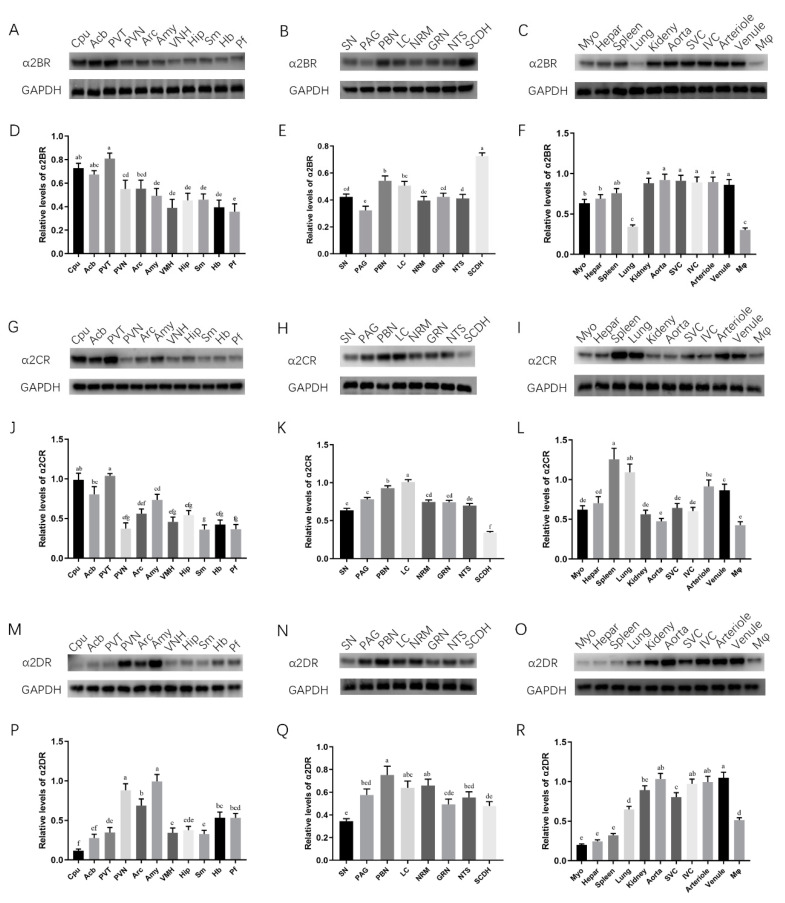
Western blot analyses of α2DR, α2BR and α2CR protein abundances in the Cpu, Acb, PVT, PVN, Arc, Amy, VMH, Hip, Sm, Hb, Pf, SN, PAG, PBN, LC, NRM, GRN, NTS, SCDH, Myo, liver, spleen, lung, kidney, aorta, SVC, IVC, arterioles, venules and Mφ of goats. (**A**–**C**) Representative bands for α2BR and GAPDH. (**G**–**I**) Representative bands for α2CR and GAPDH. (**M**–**O**) Representative bands for α2DR and GAPDH. (**D**–**F**,**J**–**L**,**P**–**R**) Densitometry analyses of protein bands. Data were normalized to GAPDH as the housekeeping protein and are presented as the means ± SD (*n* = 3). Bars with different letters differ (*p* < 0.05) Original Western blot figures are in Figure S5.

Western blot analyses were performed in this study to further confirm the relative levels of α2DR and α2BR and α2CR in goat CNS nuclei, regions and peripheral tissues. Figure 3A–C demonstrates the Western blotting results of The relative expression level was defined as the ratio of the protein level of each α2R subtype to the housekeeping protein. The pain-related nuclei that we selected were distributed in the forebrain, midbrain and hindbrain. Similar to the mRNA results, proteins for all three α2R subtypes were detected in the selected nuclei, spinal cord and peripheral tissues.

In the forebrain (Figure 3A,D,G,J,M,P), the protein levels of α2BR and α2CR were predominantly detected in the Cpu, Acb and PVN, whereas α2DR was mainly distributed in the PVT, Arc and Amy. α2CR and α2DR had relatively equal distributions in the midbrain, hindbrain and SCDH; nevertheless, α2BR was expressed at higher levels in the SCDH than in the midbrain and hindbrain. Regarding the peripheral tissues, α2CR was predominantly expressed in the spleen, lung, arteriole and venule, and a high abundance of α2DR was detected in the aorta, IVC arteriole and venule, whereas α2BR showed a relatively equal distribution. These results were generally consistent with the mRNA data and indicated that α2R subtypes are unevenly expressed in different CNS nuclei and tissues.

### 3.3. α2R-Mediated Analgesia in the Goat Spinal Cord

**Figure 4:**
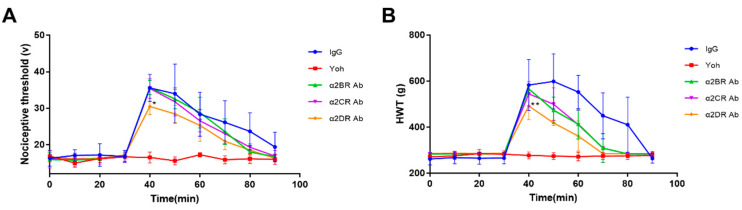
The effect of different α2R subtypes expressed in the spinal cord on the pain threshold of goats. Potassium iontophoresis (**A**) and HWT (**B**) thresholds of the tested goats represent the effect of the intrathecal injection of α2R subtype antibody on the potassium iontophoresis and HWT thresholds of goats, respectively. Data are presented as the means ± SD (*n* = 6 in each group). * *p* < 0.05 compared with the IgG group and ** *p* < 0.01 compared with the IgG group.

The characteristics of α2R-mediated analgesia in the goat spinal cord were explored by intrathecally injecting the neutralizing antibodies (Frdbio, Wuhan, China) against the a2R subtypes (30 μg/kg), nonspecific immunoglobulin (30 μg/kg) and yohimbine (3 μg/kg) in each goat according to the group (IgG, Yoh, α2BR Ab, α2CR Ab and α2DR Ab groups), followed by an intramuscular injection of xylazine (0.05 mg/kg) 30 min after the antibody injection. The potassium iontophoresis threshold and HWT threshold were recorded every 10 min, as shown in Figure 4. Yoh was used as an α2R antagonist in the control group, and neither the potassium iontophoresis threshold nor the HWT threshold changed significantly with time, as shown in Figure 4, indicating that the effect of xylazine was blocked by yohimbine.

The peak effect of xylazine occurred at 40 min in every group except for the Yoh group. The α2DR Ab group showed the lowest value for the peak effect in both Figure 4A,B, and a significant difference was not observed between the IgG group and the α2BR Ab or the α2CR Ab group (*p* > 0.05). Based on these results, α2DR expressed in the spinal cord may play a major role in α2R-mediated analgesia in goats.

### 3.4. Effect of α2Rs Expressed in the Spinal Cord on Physiological Indices of Goats

The effects of α2Rs expressed in the spinal cord on the physiological indices of goats were assessed by monitoring the physiological indicators in goats from each group before and after the intrathecal injection of antibodies.

**Figure 5:**
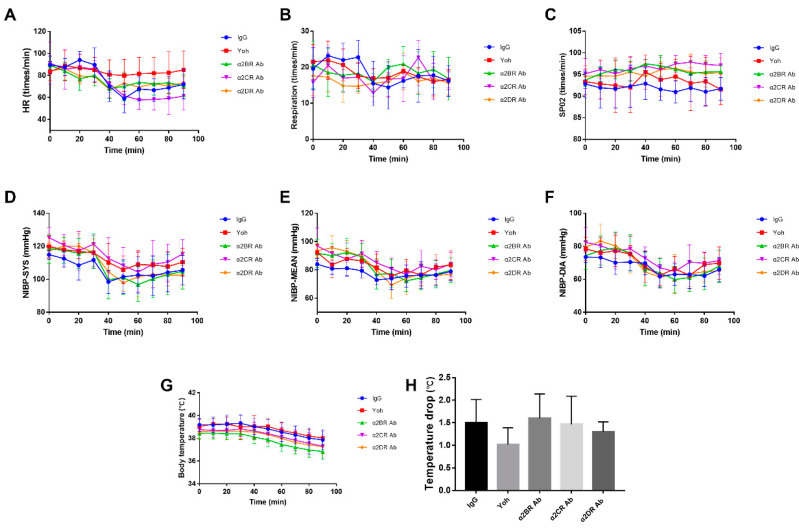
Monitoring of physiological indices in the goats from each group. (**A**–**G**) Effects of different α2R subtypes expressed in the goat spinal cord on HR, RESP, SPO2, NIBP-SYS, NIBP-MEAN, NIBP-DIA and body temperature. (**H**) Decreased body temperature in the goats of each group. Data are presented as the means ± SD (*n* = 6 in each group).

The HR decreased slightly in the IgG group and α2CR antibody group after the xylazine injection, but still fluctuated in a normal range. SPO2 generally did not show obvious fluctuations, with relatively lower values observed in the IgG and Yoh groups. NIBP-SYS, -MEAN and DIA started to decrease in all groups within 20–30 min after the xylazine injection (time 30) by no more than 20 mmHg and then began to slowly increase (Figure 5A–G). Furthermore, the temperature showed a slowly decreasing trend in all groups after the xylazine injection. The value for the decrease in temperature in each group was also calculated (Figure 5H), showing no distinct differences between different groups.

### 3.5. α2R-Mediated Analgesia in the Goat Brain

**Figure 6:**
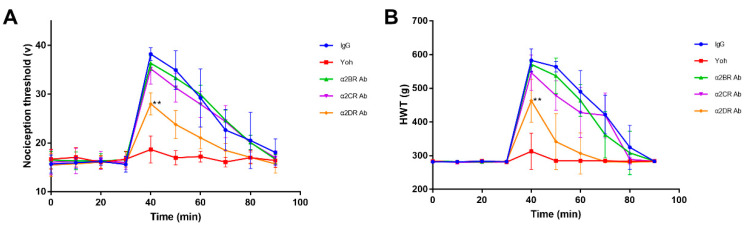
The effects of different α2R subtypes expressed in the brain on the pain threshold of goats. (**A**,**B**) Effects of the intrathecal injection of α2R subtype-specific antibodies on potassium iontophoresis and the HWT threshold of goats, respectively. Data are presented as the means ± SD (*n* = 6 in each group). ** *p* < 0.01 compared with the IgG group. 3.6 Effects of α2Rs expressed in the brain on the physiological indices of goats.

The characteristics of α2R-mediated analgesia in the goat brain were also explored with a similar method, i.e., intracerebroventricularly injecting the neutralizing antibodies against the a2R subtypes (30 μg/kg), nonspecific immunoglobulin (30 μg/kg) and yohimbine (3 μg/kg) within each goat according to the group (IgG, Yoh, α2BR Ab, α2CR Ab and α2DR Ab groups) followed by an intramuscular injection of xylazine (0.05 mg/kg) 30 min after the antibody injection. Similar results were obtained as in the spinal cord experiment. As showed in Figure 6, the peak effect of xylazine at 40 min was significantly lower in the α2DR Ab group than in the IgG, α2BR and α2CR Ab groups (*p* < 0.01), whereas no significant difference was observed between the IgG group and the α2BR or α2CR Ab group (*p* > 0.05). Thus, among the three α2R subtypes of goats, α2DR expressed in the goat brain plays a major role in the analgesic effects.

**Figure 7:**
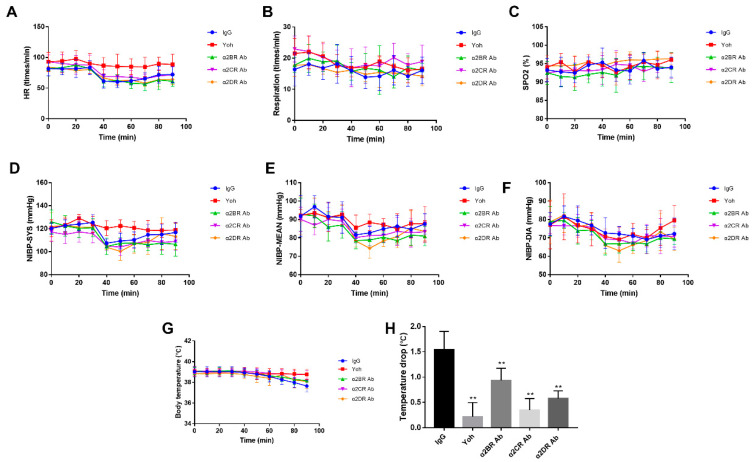
Effects of different α2R subtypes expressed in the brain on the physiological indices of goats. (**A**–**G**) Effects of different α2-AR subtypes expressed in the goat brain on HR, RESP, SPO2, NIBP-SYS, NIBP-MEAN, NIBP-DIA and body temperature. (**H**) The range of decrease in the body temperature of goats from each group. Data are presented as the means ± SD (n = 6 in each group). ** *p* < 0.01 compared with the IgG group.

We also monitored the physiological indicators in goats from each group after the intracerebroventricular injection of antibodies and xylazine to investigate the effects of α2Rs expressed in the brain on the physiological indices of the goats. The physiological indicators also included HR, RESP, SPO2, NIBP-SYS, NIBP-MEAN, NIBP-DIA and body temperature. All physiological indices monitored throughout the experiment were within the normal range and exhibited small fluctuations (Figure 7A–G).

HR started to decrease slightly after the xylazine injection in all groups except the Yoh group and remained at a lower level until the end of the experiment. No distinct changes in RESP and SPO2 were noticed in any group. IgG, α2BR, α2CR and α2DR Ab groups exhibited slight decreases in NIBP-SYS, MEAN and DIA 10 to 20 min after xylazine was injected, and then the values gradually increased after the nadir was reached. Similar to the results from the intrathecal injection experiment, all groups except the Yoh group also showed decreasing trends in these parameters. After the intraventricular injection of xylazine, the body temperature gradually and continuously decreased in all groups except the Yoh group, starting at 30 to 40 min till the end of the experiment, but the difference was insignificant (*p* < 0.01). The α2CR antibody group presented the greatest decrease, as shown in Figure 7H. Therefore, blocking α2Rs in the goat brain ameliorates the decrease in body temperature induced by xylazine, and α2BR plays a more important role in modulating body temperature.

## 4. Discussion

α2R agonists are some of the most commonly used sedative and analgesic drugs in ruminants [23]. Interestingly, the dose of α2R agonists is less than one-tenth of that in other animals, while side effects in ruminants are still very prominent [19]. The present study is the first to report the distribution and function of α2DR, α2BR and α2CR in goats. These results not only contribute to explaining the characteristics of the effects of α2R agonists on ruminants but also lay the foundation for developing α2R agonists with high α2R subtype specificity, which may increase sedative and analgesic effects and decrease side effects in ruminants.

In our study, the mRNA results were generally consistent with the Western blot results. All three α2R subtypes were detected in the goat brain, and the α2DR and α2CR subtypes were the most abundant. These results are similar to those of a previous report examining humans and rats. Harrison et al. reported that α2AR, which is the orthologue of α2DR [24], and α2CR are the dominant subtypes expressed in the brains of rats and humans [25,26]. α2AR and α2CR are both adrenoreceptors expressed in noradrenergic neurons and usually coexist in the same area in the central nervous system [27]. Interestingly, the α2DR and α2BR mRNAs were expressed at high levels in the spinal cord, in contrast to a previous report that the human spinal cord expresses both α2CR and α2BR, while the rat spinal cord predominantly expresses α2CR subtypes.

Regarding the differences in α2R subtypes at the nuclear level in the brain, the mRNA and Western blot results revealed different ratios of α2R subtypes in the different nuclei. α2AR was the dominant subtype in most nuclei tested in this study, except for the CPu, Arc, Amy, Acb, hip and PVT, where α2CR was the main subtype expressed. The Amy, AcB and Hip belong to the limbic system, and the Cpu and Arc are regions of the basal ganglia. Both the limbic system and basal ganglia are related to pain signal modulation in a chronic pain status [28]. The high expression level of α2CR in these areas makes it a potential therapeutic target for treating chronic pain in the future. In addition, the level of the α2BR mRNA was much lower than that of the α2DR and α2CR subtypes in our study. A previous report showed that α2BR expression increases in response to neuropathic pain [29]. Thus, α2BR is an inducible receptor that may play a role in pathology. Recent studies also confirmed that α2R agonists are effective treatments for neuropathic pain [30,31]; however, the role of α2R subtypes in this chronic pain disease is unclear, and related studies are still ongoing.

The distribution of α2DR, α2BR and α2CR was also investigated in peripheral tissues, such as the kidney, liver, aorta, vena cava and macrophages. Our results showed a distinct expression pattern of α2R subtypes in different areas of the vascular system. α2BR is the major subtype expressed in myocardium. α2DR is predominantly expressed in aortas and arterioles, whereas α2CR is mainly expressed in the vena cava and venules of goats. In humans, α2CR is mainly responsible for venous constriction, and α2AR is responsible for arterial constriction [32], consistent with our findings in goats. However, in dogs, α2AR is mainly detected in veins [33]. The myocardium of goats expressed mainly α2BR and α2CR in our study; however, Regitz-Zagrosek reported that the human myocardium mainly expresses α2CR [34] and that mice mainly express α2AR [35]. These results indicated species variation in the α2R distribution pattern. Notably, although the expression level of α2BR is relatively low in the nervous system, it is predominant in the liver. However, the biological function of α2BR in the liver is still unknown. A large dose of α2R agonist was reported to activate pulmonary intravascular macrophages (PIMs) and cause severe pulmonary edema in ruminants [14]. PIMs are not able to be isolated in large amounts, because they originate from peripheral mononuclear cells. We isolated mononuclear cells from the blood as a surrogate of PIMs. Mononuclear cells mainly expressed α2DR and α2CR, but did not express α2BR. Therefore, α2R agonists that are more selective for α2BR may be less likely to induce pulmonary edema in the ruminant. In addition, a balanced anesthesia could decrease the dosage of alpha2 agonist and reduce pulmonary edema in clinical practice.

The injection of alpha 2 adrenergic receptor agonists such as xylazine will exert certain side effects on goats, such as a lower body temperature, excessive salivation and an initial increase in blood pressure followed by a decrease. In the α2CR antibody intracerebroventricular injection group, the body temperature of goats did not decrease significantly 90 min after the α2CR antibody injection, while no difference in body temperature was observed between the α2CR antibody intrathecal injection group and other five groups. Based on this result, α2CR activation exerts a greater effect on body temperature than α2BR and α2DR activation. The thermoregulation center is located in the hypothalamus, and the insignificant decrease in temperature after the injection of α2CR antibody may be due to the low expression of α2CR in the hypothalamus. In our experiment, the hypothalamic nuclei PVN, Arc and VMH expressed α2CR at lower levels than the other two receptors, which also supports this result to a certain extent. However, the blood pressure of the goats did not first increase and then decrease in the experiment. Since the effect of α2R agonists on blood pressure is related to its dose [36] and the dose of xylazine used in the experiment was lower than the clinical dose, biphasic blood pressure changes in goats could not be detected.

After the xylazine injection, the pain threshold of potassium iontophoresis and HWT increased and peaked after 10 min. By comparing the pain thresholds of each group at this time point, we found that blocking α2DR in both the intrathecal injection experiment and the intracerebroventricular injection experiment antagonized the effect of xylazine on the pain threshold, while no significant difference in the effects of the antagonists of the other two subtypes were observed compared with the IgG group. Based on our previous research, we selected 11 pain-related nuclei in the forebrain, 2 in the midbrain and 5 in the hindbrain, and measured the distribution and expression of different subtypes of α2R. Among them, the PAG–Cpu–Amy–Hb–PAG constitutes the limbic analgesia circuit. The PAG–rostral ventromedial medulla (RVM)–dorsal horn of the spinal cord circuit is the main part of the brainstem descending inhibitory pathway [37]. The Sm–ventral lateral orbitofrontal cortex (VLO)–PAG pathway has also been identified. These three pathways play important roles in regulating pain or nociception. Combined with the results of the antibody injection test, the expression of α2DR in the PAG, NRM, GRN, NTS and SCDH, which play important roles in the descending inhibitory pathway of pain, was higher than that of the other two α2R subtypes. Similarly, the PAG, Amy and Hb, which play important roles in a mesolimbic loop of analgesia, expressed α2DR at higher levels than the other two α2R subtypes. The Sm, which plays an important role in the Sm–VLO–PAG pathway, displayed a significantly higher expression of α2DR than the other two α2R subtypes. We inferred that α2DR plays a major role in α2R-mediated analgesia in goats. Therefore, α2DR-selective agonists may exert better analgesic effects on ruminants.

## 5. Conclusions

In summary, we demonstrate the presence and heterogeneity of α2R subtype mRNA and protein in different nuclei of the goat brain, selected regions of the spinal cord (L4-L6) and various peripheral tissues. We also demonstrate the functional characteristics of α2BR, α2CR and α2DR in goats. Our study revealed that α2DR is more essential for α2R-mediated analgesia, whereas α2CR activation exerts a greater effect on body temperature. Such information enables the development of α2R agonists with high α2R subtype specificity, which may increase sedative and analgesic effects and decrease side effects in ruminants. In addition, the elucidation of the relationship of a2R subtypes to CNS pathways will be important in understanding the mechanisms of nociception, autonomic function and muscle tone at the CNS level.

## Data Availability

The data presented in this study are available on request from the corresponding author.

## Supplementary Materials

The following supporting information can be downloaded at: https://www.mdpi.com/article/10.3390/ani12050664/s1, Figure S1: Amino acid sequence alignment of goat α2-adrenergic receptor subtypes. α2B, α2C and α2D subtypes; Figure S2: Amino acid sequence alignment of human (NP_000672.3), mouse (NP_031443.4), cattle (NP_776924.1), and goat (XP_017897206.1) α2A/D-adrenergic receptor subtypes; Figure S3: Amino acid sequence alignment of human, mouse, cattle, and goat α2B-adrenergic receptor subtypes; Figure S4: Amino acid sequence alignment of human, mouse, cattle, and goat α2C-adrenergic receptor subtypes; Figure S5: Original western blot figure in Figure 3.

## Abbreviations

α2BR Ab	α2BR antibody;
α2CR Ab	α2CR antibody;
α2DR Ab	α2DR antibody;
Acb	nucleus accumbens;
Amy	amygdala;
Arc	arcuate nucleus;
Cpu	caudate putamen nucleus;
GRN	gigantocellular reticular nucleus;
Hb	habenular nucleus;
Hip	hippocampus;
HWT	hindlimb withdrawal threshold;
IgG	nonspecific immunoglobulin;
IVC	inferior vena cava;
LC	locus coeruleus;
Mφ	blood mononuclear macrophages;
Myo	myocardium;
NRM	nucleus raphe magnus;
NTS	nucleus of the solitary tract;
PBN	parabrachial nucleus;
Pf	parafascicular nucleus;
PAG	periaqueductal gray;
PVN	hypothalamic paraventricular nucleus;
PVT	paraventricular thalamus;
SCDH	spinal cord L4-L6 dorsal horn;
Sm	thalamic nucleus submedius;
SN	substantia nigra;
SVC	superior vena cava;
VMH	ventromedial hypothalamic nucleus;
Yoh	yohimbine
