# Supplementary material for: The Characteristics and Distribution of α2D-, α2B- and α2C-Adrenoceptor Subtypes in Goats
Source: Animals (Basel). 2022 Mar 7;12(5):664. doi: 10.3390/ani12050664 (PMC8909296; doi:10.3390/ani12050664)
Supplement: Supplementary file 1 [file animals-12-00664-s001.zip › animals-1555005-supplementary.pdf]

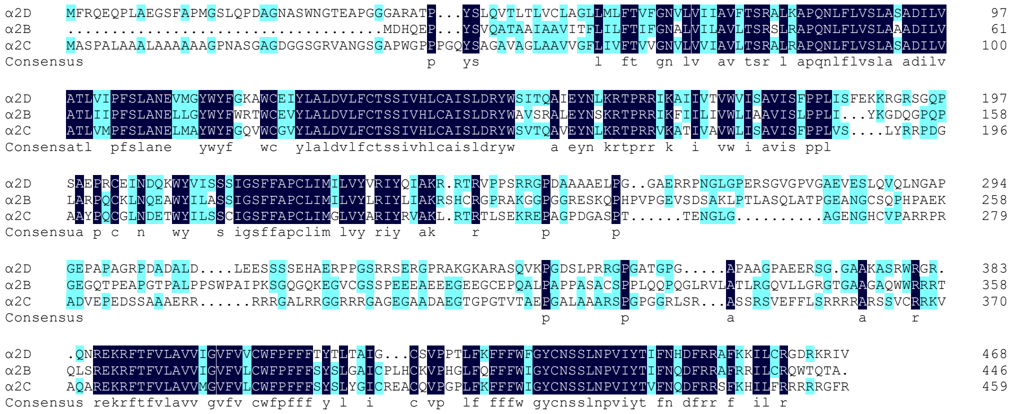

Supplementary figure S1. Amino acid sequence alignment of goat α2-adrenergic receptor subtypes. α2B, α2C and α2D subtypes.

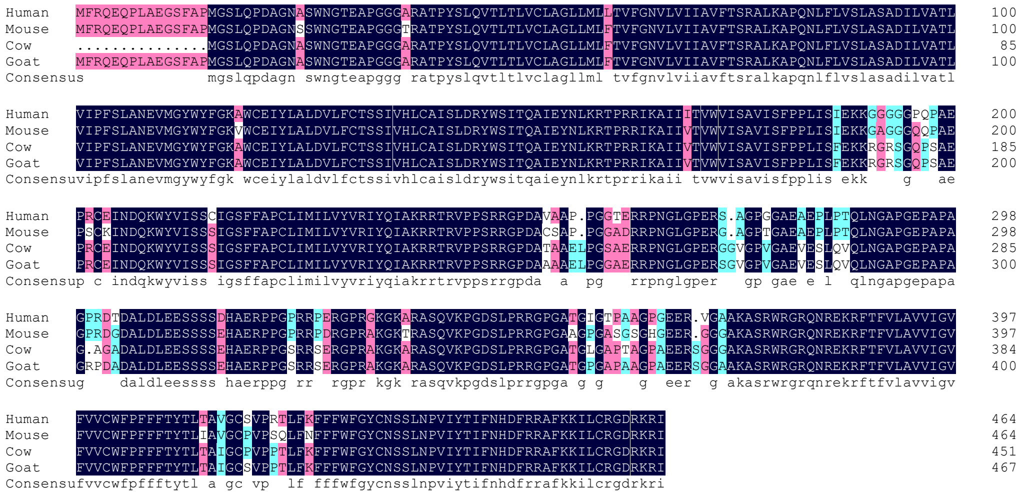

Supplementary figure S2. Amino acid sequence alignment of human (NP_000672.3), mouse (NP_031443.4), cattle (NP_776924.1), and goat (XP_017897206.1) α2A/D-adrenergic receptor subtypes.

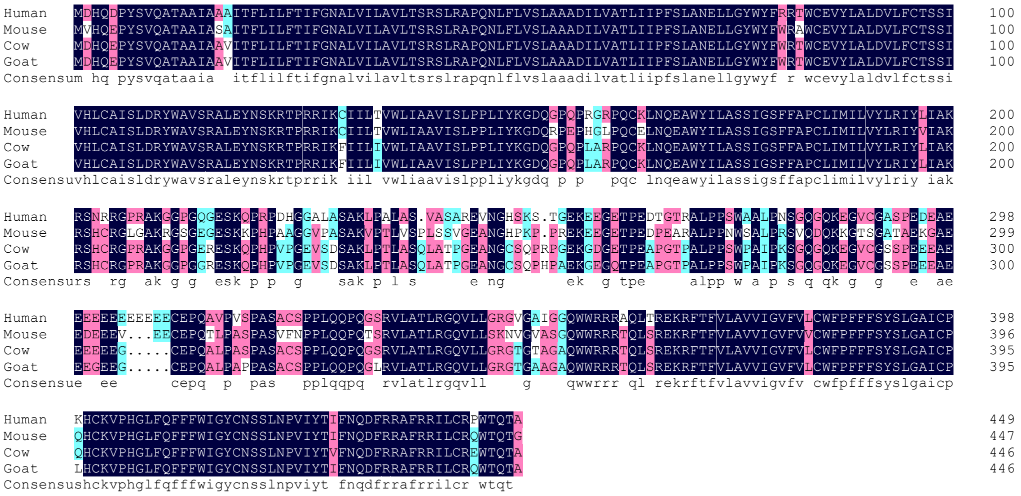

Supplementary figure S3. Amino acid sequence alignment of human, mouse, cattle, and goat α2B-adrenergic receptor subtypes.

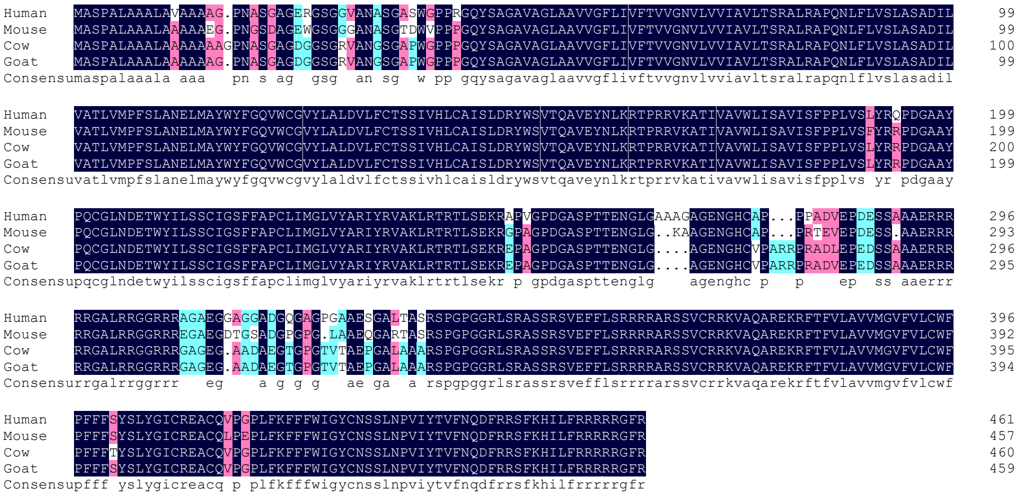

Supplementary figure S4. Amino acid sequence alignment of human, mouse, cattle, and goat α2C-adrenergic receptor subtypes.

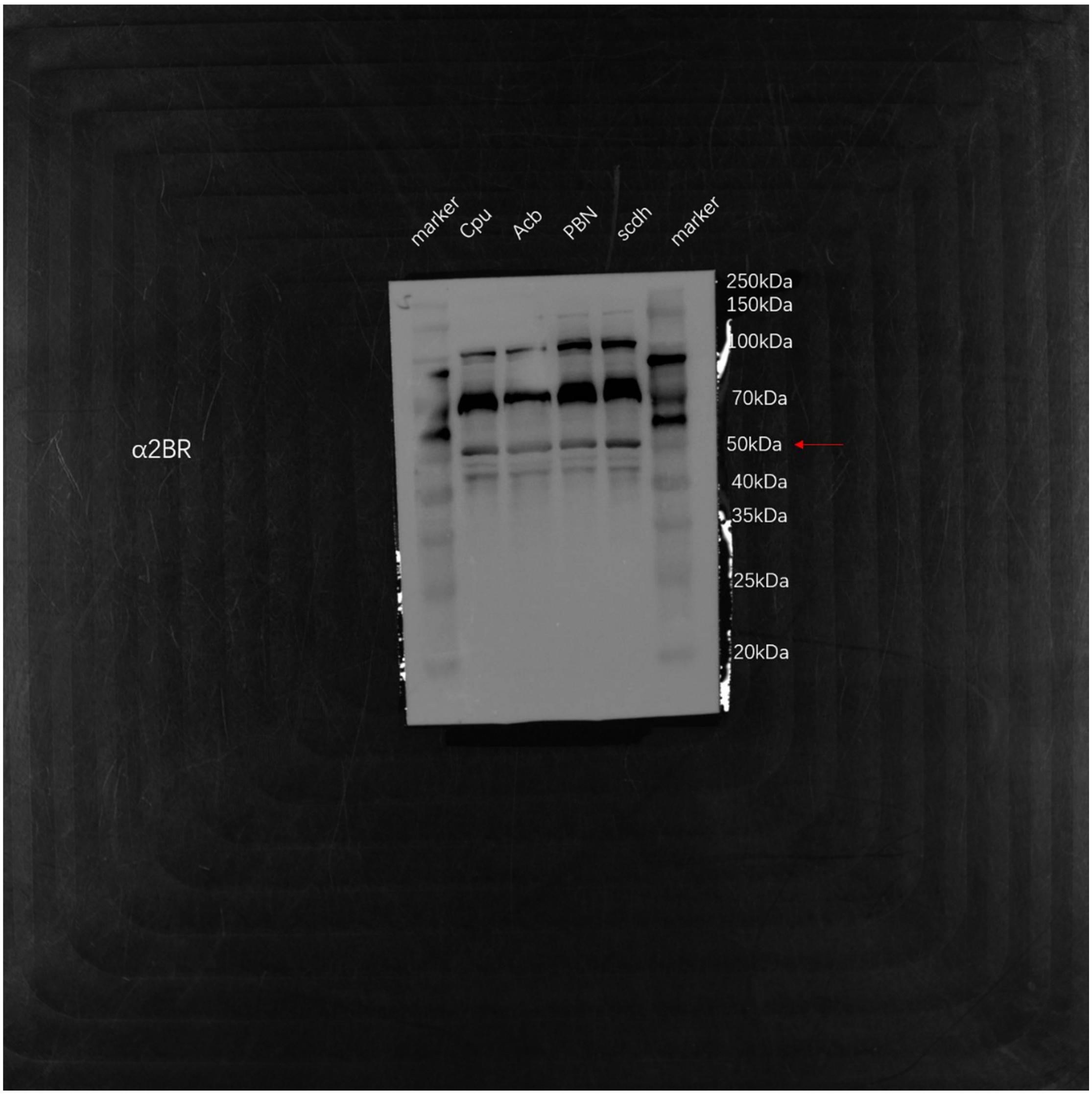
α2BR
marker
Cpu
Acb
PBN
scdh
marker
250kDa
150kDa
100kDa
70kDa
50kDa
40kDa
35kDa
25kDa
20kDa

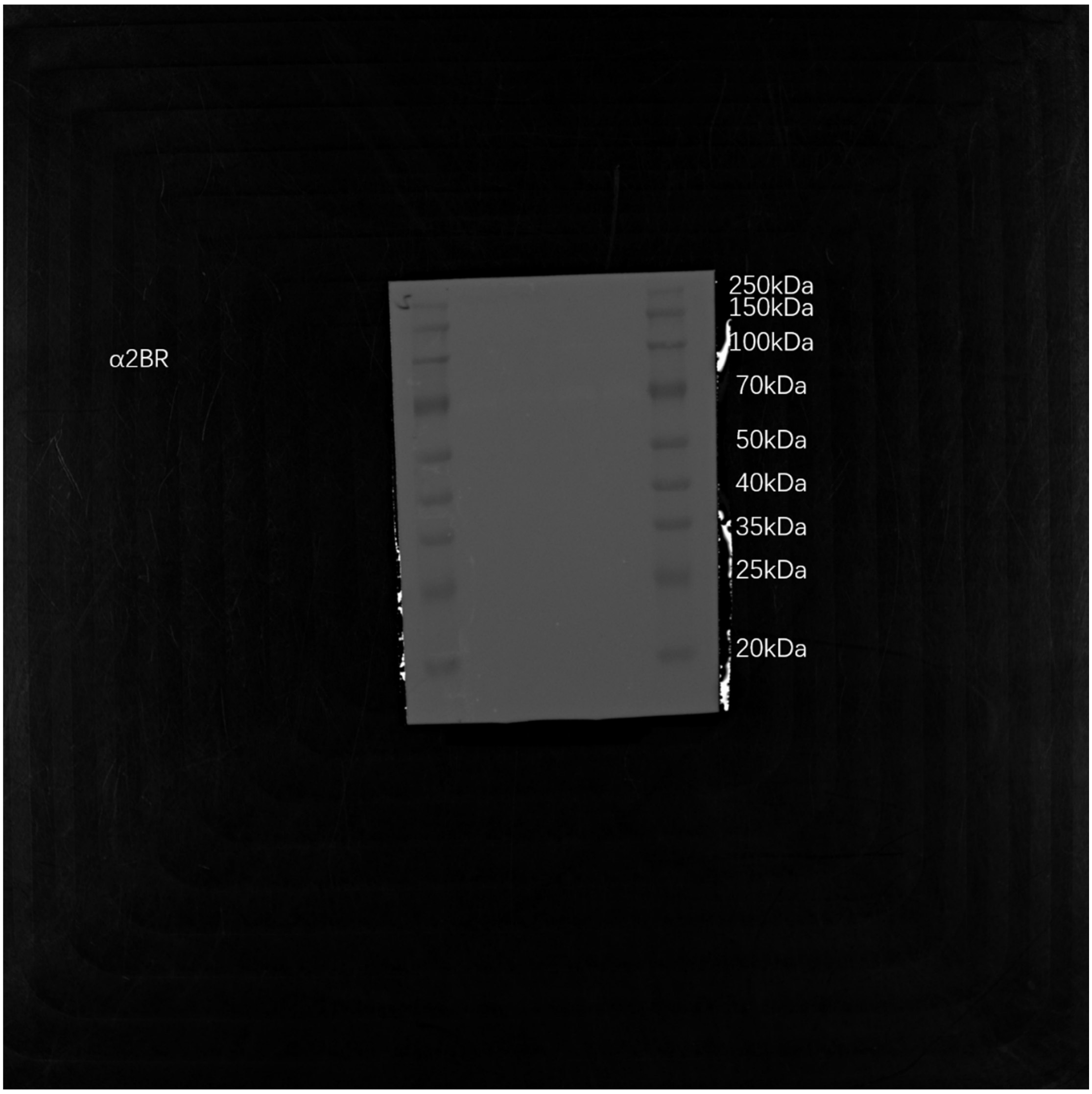
α2BR
250kDa
150kDa
100kDa
70kDa
50kDa
40kDa
35kDa
25kDa
20kDa

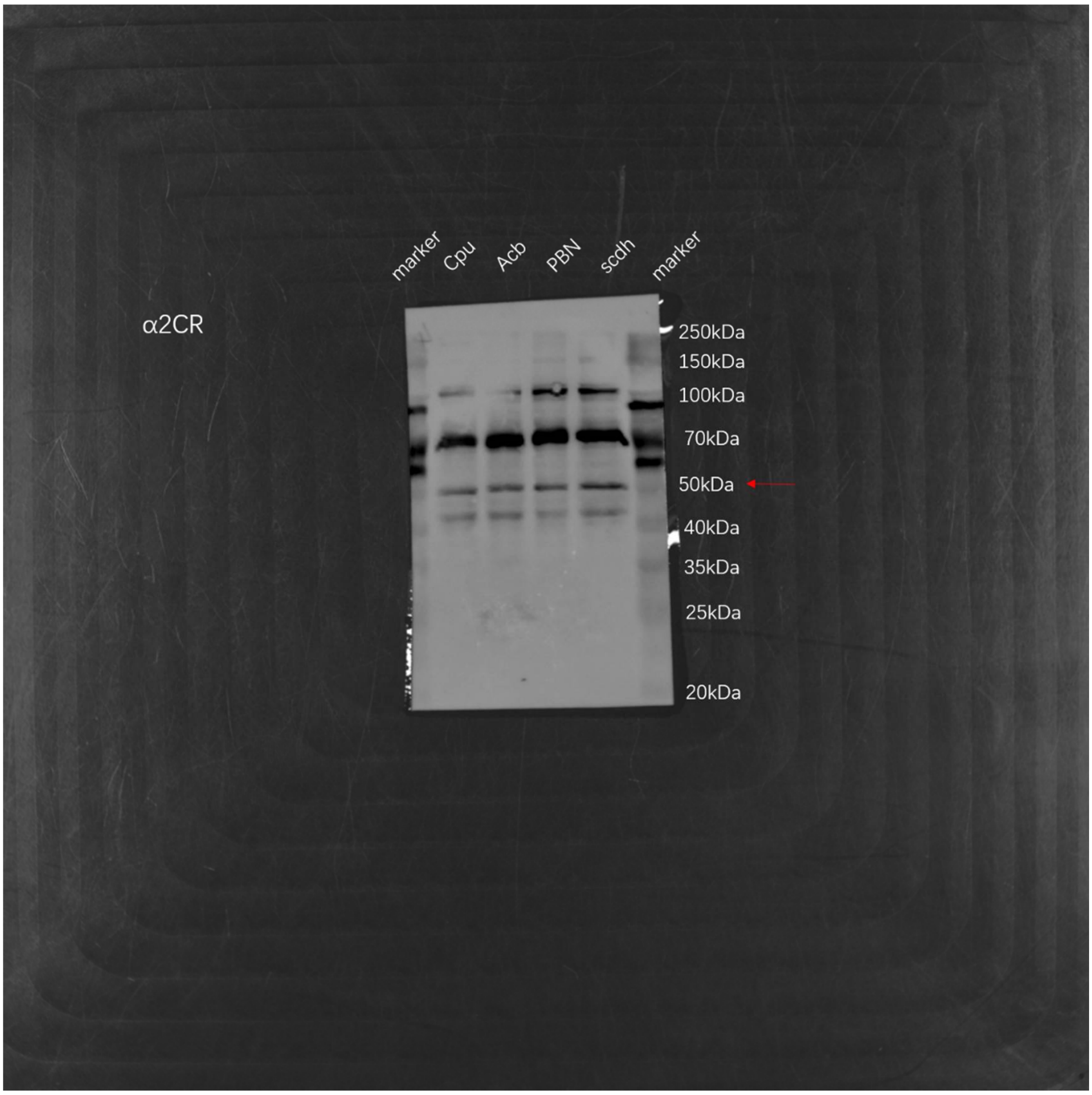
α2CR
marker
Cpu
Acb
PBN
scdh
marker
250kDa
150kDa
100kDa
70kDa
50kDa
40kDa
35kDa
25kDa
20kDa

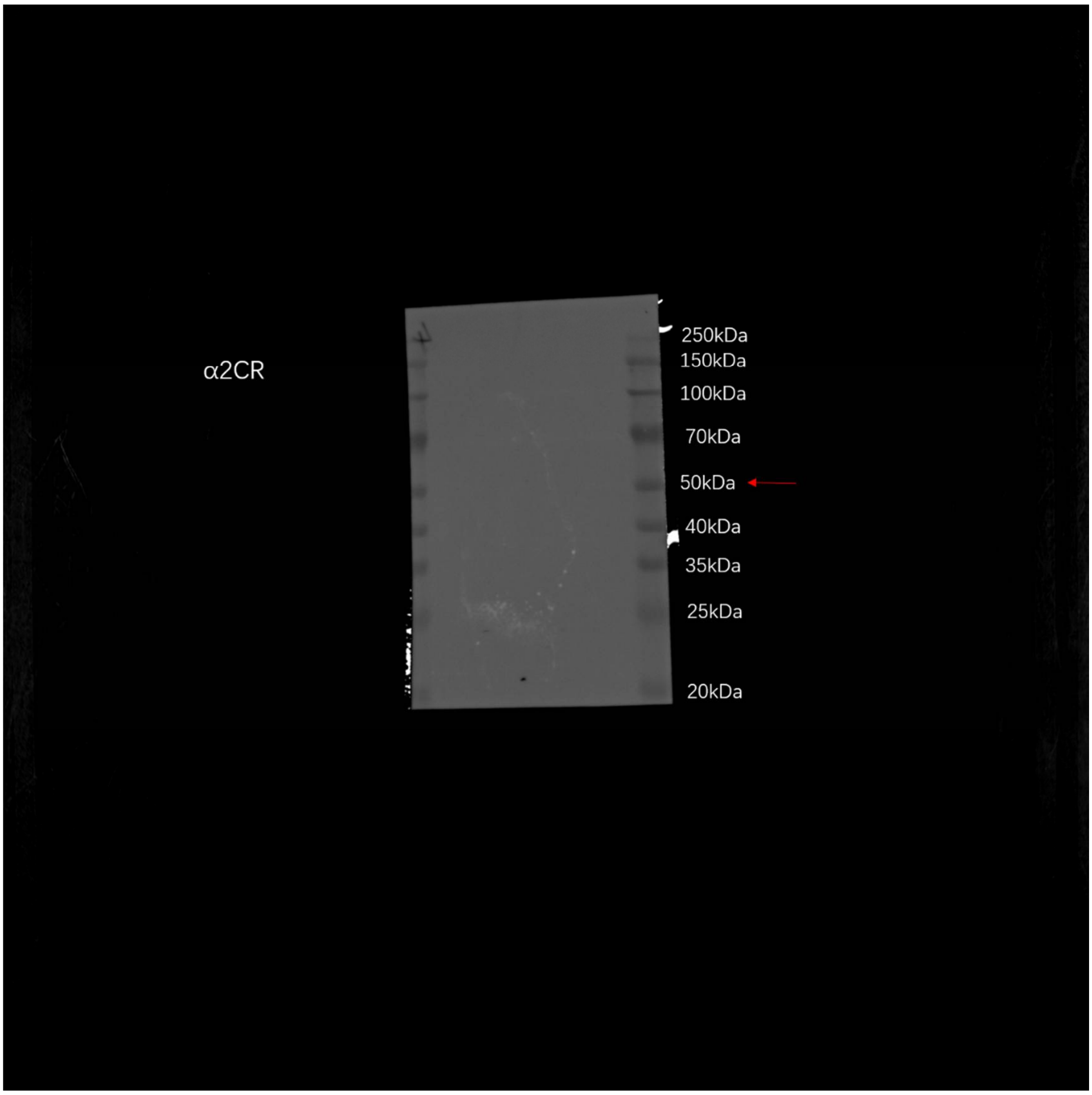
α2CR
250kDa
150kDa
100kDa
70kDa
50kDa
40kDa
35kDa
25kDa
20kDa

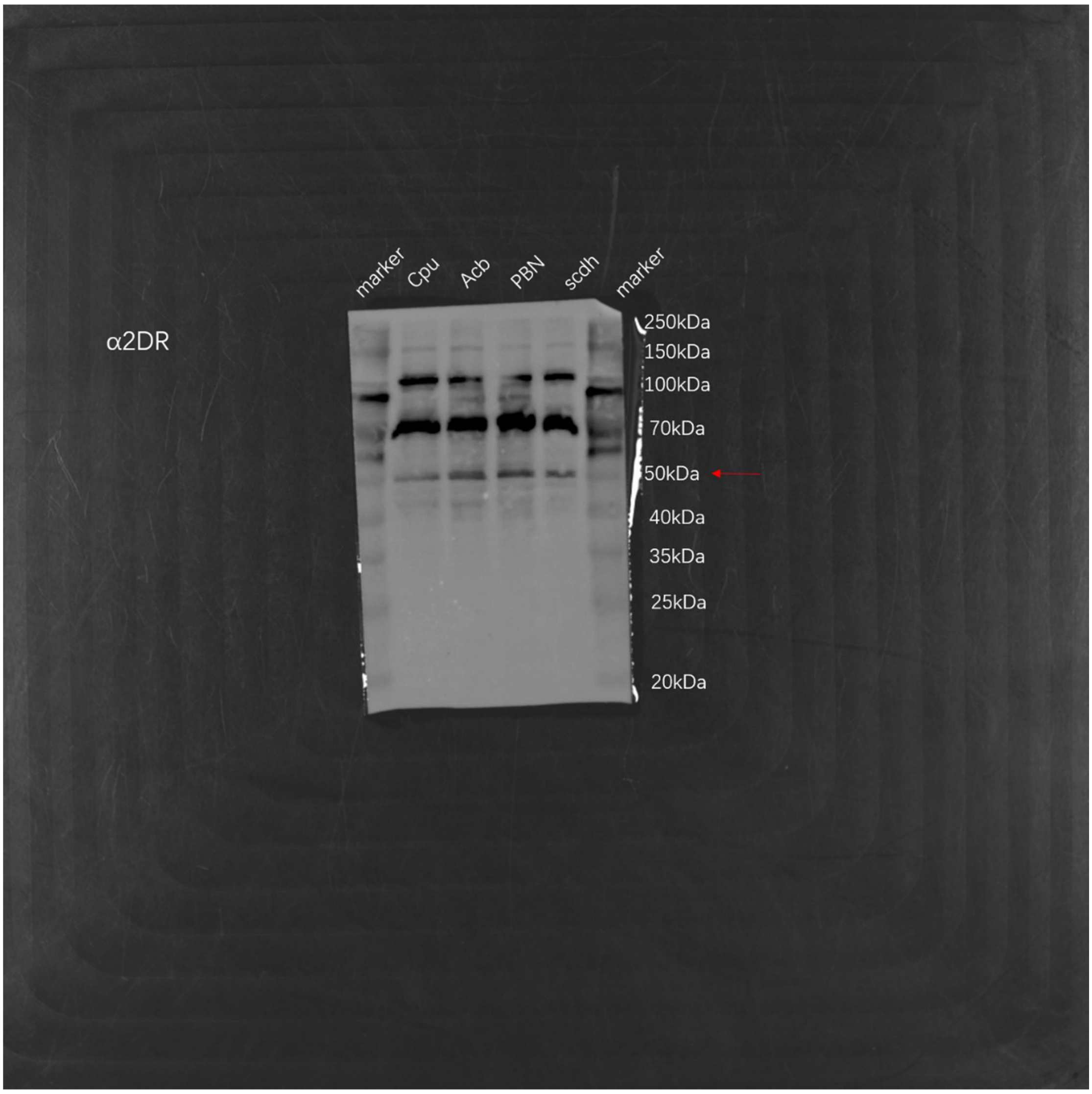
α2DR
marker
Cpu
Acb
PBN
scdh
marker
250kDa
150kDa
100kDa
70kDa
50kDa
40kDa
35kDa
25kDa
20kDa

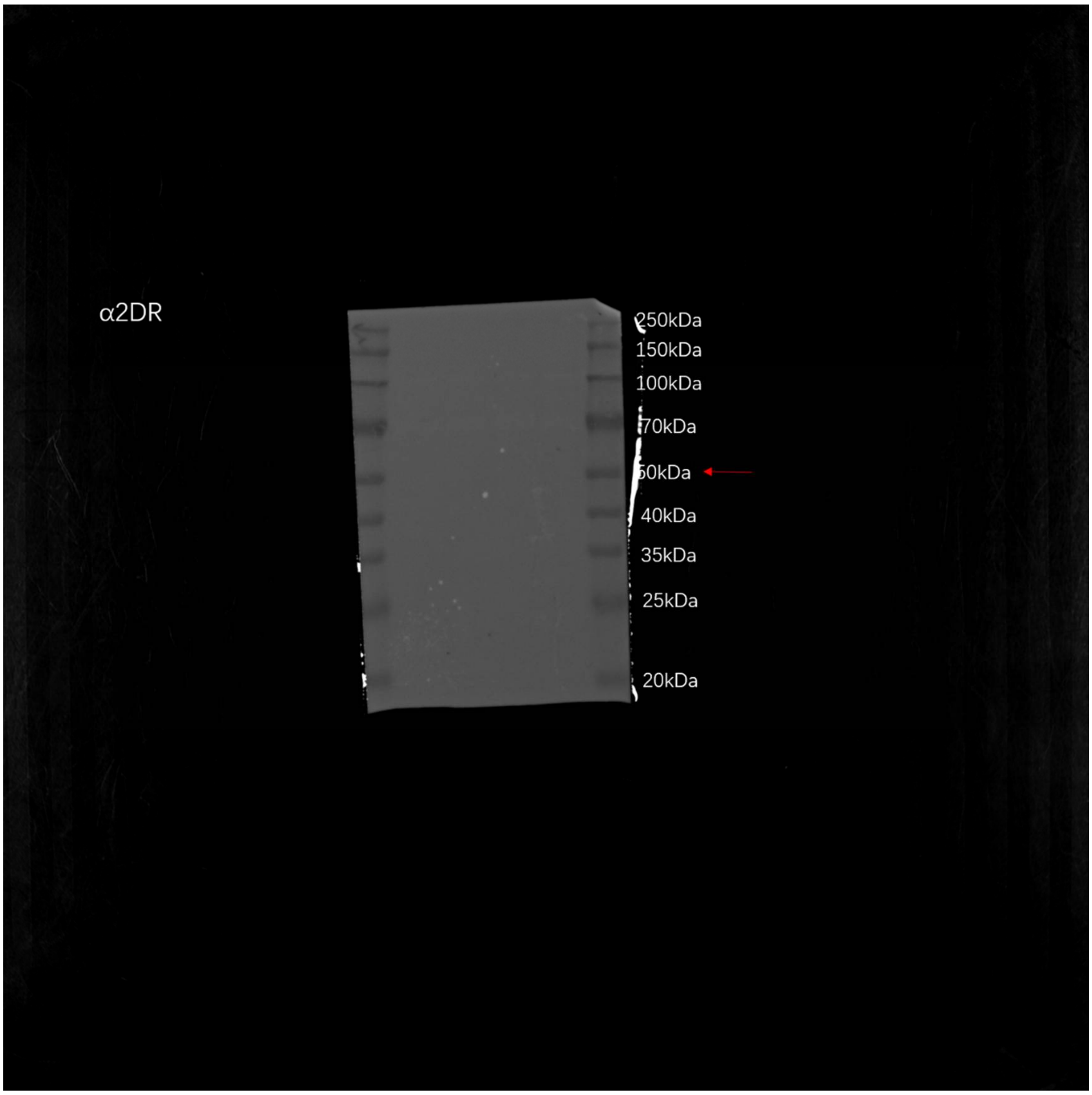

Supplementary figure S5. Original western blot figure in Figure 3
